# Pneumococcal serotype evolution in Western Europe

**DOI:** 10.1186/s12879-015-1147-x

**Published:** 2015-10-14

**Authors:** Myint Tin Tin Htar, Dina Christopoulou, Heinz-Josef Schmitt

**Affiliations:** Pfizer Vaccines, Medical Development Group and Scientific Affairs, 23-25 avenue du Dr. Lannelongue, F-75668 Paris, Cedex 14 France

**Keywords:** Invasive pneumococcal diseases, Pneumococcal serotype, Pneumococcal conjugate vaccines

## Abstract

**Background:**

Pneumococcal diseases remain a leading cause of vaccine-preventable death worldwide in children <5 years of age. The seven-valent pneumococcal conjugate vaccine (PCV7) was approved in 2001 in Europe and was introduced into the national immunization programmes of many European countries from 2006–2008. In 2009, higher-valent PCVs (PCV10 and PCV13) became available, replacing PCV7 from 2009–2011. This article describes the evolution of vaccine and non-vaccine serotypes causing invasive pneumococcal disease (IPD) following the introduction of PCVs in Western Europe, based on data from publicly-available medical publications and national surveillance systems from January 2010 to May 2015.

**Discussion:**

In countries with high vaccine uptake, 5–7 years after PCV7 introduction IPD caused by vaccine serotypes has almost disappeared in children. Non-PCV7 serotypes have emerged, particularly serotypes 19A, 7 F, 3 and 1. A rapid and significant reduction of the additional serotypes included in higher-valent vaccines has been observed consistently following the introduction of these vaccines. A significant and rapid decline of serotypes 19A, 7 F, 1 and 6A in both vaccine-eligible and older age groups has been observed in countries using PCV13 while serotype 19A and 3 has increased in countries using PCV10. Serotype 3 has become one of the most prevalent serotypes in adults, with some reduction only in the UK and France. Serotype diversity increased and varied by age group, the type of vaccine in use, and the time since the introduction of higher-valent PCVs. Serotypes that are currently more frequent include 24 F, 22 F, 8 and 15A in countries that use PCV13, and serotypes 19A and 3 in countries that use PCV10. Compared with the time before the introduction of higher valent PCVs, to date, there is no single ‘19A-like’ serotype emerging across countries and most of the newly emerging non-PCV13 vaccine types are less invasive with a low case-carrier ratio.

**Conclusions:**

It is important to closely monitor not only evolving serotypes but also the magnitude of the effect in order to evaluate the overall impact of pneumococcal vaccination programmes and to initiate the appropriate vaccination strategy. Emerging serotypes may also need to be considered for the future development of new vaccines.

## Background

Pneumococcal diseases remain a leading cause of vaccine-preventable death worldwide in children <5 years of age [1]. The first pneumococcal conjugate vaccine (PCV7) comprising serotypes 4, 6B, 9 V, 14, 18C, 19 F, and 23 F was licensed in 2001 in Europe for the prevention of pneumococcal disease in children. It was introduced into the national immunization programmes (NIPs) of many European countries between 2006 and 2008. In 2009, higher-valent PCVs became available: PCV10 (including additional serotypes 1, 5 and 7 F) and, later PCV13 (including additional serotypes 1, 3, 5, 6A, 7 F and 19A) [2]. These vaccines usually replaced PCV7 between 2009 and 2011.

PCV immunization has been successful in decreasing the mortality and morbidity associated with invasive and mucosal pneumococcal diseases caused by vaccine serotypes in children [3–8]. A reduction in nasopharyngeal carriage of vaccine serotypes has also led to a substantial herd effect (indirect protection) in the older population [9]. Following the introduction of PCV7, a marked reduction in diseases related to vaccine serotypes has been observed alongside the emergence of non-vaccine serotypes [6, 10]. This article describes the evolution of vaccine and non-vaccine serotypes causing invasive pneumococcal disease (IPD) following the introduction of PCVs in Western Europe.

Data are included from national surveillance systems as well as representative observational studies available within Medline publications and national surveillance websites from January 2010 to May 2015 in 15 Western European countries: Austria, Belgium, Denmark, Finland, France, Germany, Ireland, Italy, The Netherlands, Norway, Portugal, Spain, Sweden, Switzerland and the UK. Single-centred observational studies or clinical trials were not considered for this review. Only data from original articles were considered.

## Discussion

### Countries in which only PCV13 is used

#### Belgium

PCV7 was introduced into the NIP in January 2007 with a 2 + 1 schedule and was replaced by PCV13 in July 2011. By 2012, the vaccination coverage was estimated at 98 % for the first dose and 93 % for the third dose [11].

After 4 years of implementation of PCV7 into the NIP followed by 2 years of implementation of PCV13, the overall incidence of IPD in children <2 years of age decreased continuously from 155.9 to 56.0/100,000 population between 2002–2003 and 2013, respectively (Table 1). The incidence also decreased in those 2–5 years of age but remained the same in those 5–15 years of age.

**Table 1:** Incidence of IPD (/100,000 population) in Western European countries

	Pre-PCV7 period	Post-PCV7 period	Post-higher PCV period
Countries in which only PCV13 used			
Belgium [11]	2002–2003		2013
<2 years	155.9		56.0
Denmark [14]	2000–2007	2008–2010	2011–2013
<2 years	55.1	25.9	16.0
≥65 years	65.5	60.0	49.4
France [20]	2001–2002	2008–2009	2012
<2 years	30.3	24.6	17.2
>64 years	27.7	31.1	26.5
Ireland [21]	2008	2011	2012
All ages	9.5	7.6	7.6
Norway [25]	2005	2010	2012
<2 years	77.0	20.0	8.1
All ages	23.0	15.0	13.0
Spain (Madrid) [27]	-	2007–2010	2011–2012
<15 years	-	17.1	7.7
Switzerland [29, 30]	2002–2005	2010	2012
<2 years	27.1	20.6	8.1
≥65 years	42.1	35.2	35.7
UK [31]		2008–2010	2013–2014
<2 years	-	22.2	12.0
≥65 years	-	27.6	20.6
Countries in which only PCV13 used			
Finland [39]	2006	2010	2014
<1 year	68.5	50.7	11.2
>64 years	31.1	32.3	32.5
Countries in which both PCV13 and PCV10 used			
Germany [42–44]	1997–2001	2010	2012
<2 years^a^	7.7	4.1	3.7
<2 years^b^	12.7	9.4	6.3
Spain (Navarra) [47]	2001–2003	2004–2009	2010–2013
<5 years	87.1	60.7	18.7
≥65 years	33.6	32.5	25.0
Portugal [48]		2008–2009	2011–2012
<1 year	-	52.1	25.1
1-2 years	-	31.6	16.1

^a^Pneumococcal meningitis: ^b^pneumococcal non-meningitis IPD

In 2010, following the decrease in PCV7-serotype IPD, non-vaccine-type IPD emerged. At this time, PCV13 serotypes accounted for 73 % of all IPD cases and the most prevalent serotypes were 19A, 1 and 7 F (60 % of all IPD cases). In 2013, PCV13 serotypes accounted for 36 % of IPD cases and serotypes 19A, 1 and 7 F accounted for 29 % of all IPD cases. Serotypes 12 F and 24 F became more prevalent, representing 12 and 7 % of all IPD cases, respectively. Serotype 3 was not observed in children <5 years of age in 2013 (Table 2) [11]. In 2010, PCV7 serotypes decreased significantly among individuals ≥60 years of age, and non-PCV7 serotypes (e.g., 38, 7, 12 and 22) increased [12].

**Table 2:** Prevalence of PCV13 serotypes in Western European countries in children <5 years of age. For details on local vaccine use, please see text

	Age (year)	Post-PCV7 period		Three most prevalent serotypes	Post-higher valent PCV periods		Three most prevalent serotypes
		Year	Among all IPD, %		Year	Among all IPD, %	
Countries in which only PCV13 used							
Belgium [11]	<5	2010	73	19A, 1, 7 F	2013	36	1, 12 F, 24 F
France [20]	<2	2009	71	19A, 7 F, 1	2012	24	24 F, 12 F, 15B/C
Italy [24]	<5	2011	64	1, 19A, 22 F	2014	37	19A, 1, 24 F
Norway [25]	<5	2010	61	19A, 1, 7 F	2012	26	1, 15B/C
Switzerland [29]	<2	2010–2011	68	3, 19A, 7 F	2012	53	3, 23 (not 23 F), 19 F
UK [31]	<5	2008–2010	68	7 F, 19A, 1	2013–2014	15	24 F, 22 F, 33 F
Countries in which only PCV10 used							
Austria [33, 37]	<5	2009	73	3, 14, 18C	2013	93	19A, 7 F, 23 F
Finland [39]	<5	2010	92	14, 6B, 19 F	2014	70	19A, 3, 7 F, 23A
Countries in which both PCV13 and PCV10 used							
Germany^a^ [44]	<16	2008–2009	56	7 F, 1, 18C^b^	2012–2013	12	7 F, 10A, 19A^c^
Sweden [45]	<2	2009	84	22 F, 7 F, 3, 19A	2012	47	33 F, 11

^a^Pneumococcal meningitis; ^b^2007–2010; ^c^2010–2013

#### Denmark

PCV7 was introduced into the NIP with a 2 + 1 schedule in October 2007. It was replaced by PCV13 in April 2010 with a two-dose catch-up for children 12–17 months of age. Vaccine coverage for one dose of PCV13 was 92 % in 2011 [13].

In children <2 years of age, the incidence of PCV7-serotype IPD was significantly reduced from 36.4/100,000 population in 2000–2007 to 3.9/100,000 population in 2008–2010 and to 0.3/100,000 population in 2011–2013, with only one case caused by 19 F in 2011. The overall IPD was reduced from 55.1/100,000 population to 16.0/100,000 population from 2000–2007 to 2011–2013 (Table 1) [14]. In children <3 months of age, PCV7-serotype disease has not been detected since 2010 [15]. In adults ≥65 years of age, a similar reduction was also observed for overall IPD and PCV7-serotype IPD. The incidence of PCV7-serotype IPD significantly decreased from 27.1/100,000 population in 2000–2007 to 3.3/100,000 population in 2011–2013 [14].

In 2008–2010, the most prevalent serotypes were 1, 7 F and 19A in children <5 years of age and 1, 7 F and 3 in those ≥65 years of age [14, 16, 17]. In 2013, serotype 19A significantly increased in all ages following the introduction of PCV7 but significantly decreased following PCV13 introduction (zero cases in children <2 years of age). Incidence of serotypes 1 and 3 showed no significant changes beyond the expected natural cyclical variations. Non-PCV13 serotypes accounted for nearly 80 % of cases with no clear predominance of a specific serotype in 2011–2013 [14].

#### France

PCV7 was introduced into the NIP in June 2006 with 2 + 1 schedule, and was replaced by PCV13 in June 2010 [18]. Vaccine coverage was 94 % in 2009 and remained high in 2012 [19].

Following the introduction of PCV7, the overall incidence of IPD gradually reduced in children <2 years of age from 30.3/100,000 population in 2001–2002, to 24.6/100,000 population in 2008–2009 and 17.2/100,000 population in 2012. In adults >64 years of age, the incidence increased after the introduction of PCV7 from 27.7 to 31.1/100,000 population (2001–2002 to 2008–2009, respectively), then decreased in 2012 to 26.5/100,000 population, similar to the incidence before the introduction of PCVs (Table 1).

The incidence of PCV7 serotypes significantly declined following the introduction of PCV7, and in 2012, PCV7 serotypes accounted for 6 % of all IPD cases in children <2 years of age and 8 % of cases in adults ≥50 years of age. In 2009, PCV13 serotypes accounted for 71 % of all IPD cases in children <2 years of age, which subsequently declined to 24 % in 2012 (Table 2), mainly due to a significant reduction in serotypes 19A (83 % reduction), 7 F (77 % reduction), 1 (96 % reduction) and 3 (85 % reduction). The incidence of non-PCV13 serotypes 24 F and 12 F increased, accounting for 20 and 10 % of all IPD cases, respectively. In adults >65 years of age, despite a significant decrease, PCV13 serotypes remained prevalent (42 % of all IPD cases) with 19A, 3 and 7 F predominating (Table 3) [18, 20].

**Table 3:** Prevalence of PCV13 serotypes in Western European countries in older populations

	Age (year)	Post-PCV7 period		Three most prevalent serotypes	Post-higher valent PCV periods		Three most prevalent serotypes
		Year	Among all IPD, %		Year	Among all IPD, %	
Countries in which only PCV13 used							
France [18, 20]	≥65	2009	61	19A, 7 F, 3^a^	2012	42	19A, 3, 7 F^a^
Norway [25]	>5	2010	52	7 F, 19A, 1, 3	2012	45	7 F, 19A, 3
Switzerland [29]	>64	2010–11	66	3, 19A, 7 F	2012	62	3, 19A, 7 F
UK [31]	≥65	2008–10	54	19A, 3, 22 F	2013–14	21	8, 22 F, 3
Countries in which only PCV10 used							
Austria [33, 37]	>50	2009	69	3, 6A, 14	2013	59	3, 14, 19A
Finland [39]	>65	2010	72	14, 3, 23 F	2014	60	3, 22 F, 19A
Netherlands [41]	All ages	2010	60	7 F, 8, 19A, 1	2013	45	8, 7 F, 19A
Countries in which both PCV13 and PCV10 used							
Germany^a^ [42, 44]	>16	2008–2009	56	3, 7 F, 22 F^b^	2012–2013	25	23B, 3, 7 F^c^

^a^Pneumococcal meningitis; ^b^2007–2010; ^c^2010–2013

#### Ireland

PCV7 was introduced into the NIP in September 2008 with a 2 + 1 schedule and was replaced by PCV13 in December 2010. Vaccine coverage was 91 % in 2012 for three doses in children < 2 years of age [21].

The overall incidence of IPD in general population decreased from 9.5/100,00 population in 2008 to 7.6/100,000 population in 2011 and remained the same in 2012 (Table 1). In 2008, serotype 14 was the most predominant serotype followed by 4, 9 V, 7 F, 8, 23 F and 6B. These serotypes combined accounted for approximately 53 % of all IPD cases within the population. In 2010, PCV7-serotype IPD declined by 91 % compared with 2008 in children <2 years of age and by 50 % in those >2 years of age. PCV13 serotypes accounted for 55 % of all IPD cases in all ages at the time PCV13 was introduced. In 2014, the predominant serotypes were 7 F, 15A, 22 F in all ages [21, 22].

#### Italy

PCV7 was introduced into immunization programme in May 2005 as a 2 + 1 schedule and it was replaced by PCV13 in the summer of 2010. Vaccine coverage differed between regions and was 50–60 % between 2005 and 2007 [23]. The number of IPD cases reported to the national laboratory surveillance centre was generally low but did decrease. In 2005, PCV7 serotypes accounted for 50 % of all IPD cases in children 0–4 years of age, declining to 31 % of cases in 2007 and 19 % of cases in 2011 (one case each for 23 and 19 F). In 2011, PCV13 serotypes accounted for 64 % of all IPD cases, which declined to 37 % in 2014 (Table 2). Between 2007 and 2009, two-thirds of pneumococcal bacteraemia cases in children were due to non-PCV7 serotypes; serotype 1 was the most frequent and was associated with complications, followed by serotype 19A, which was associated with younger age. Serotypes 19A and 1 were the most prevalent in 2011 and remained high in 2014 [24].

#### Norway

PCV7 was included into the NIP with a 2 + 1 schedule in July 2006, with a catch-up campaign for children born in 2006 only, and was replaced by PCV13 in April 2011. Vaccine coverage was 92 % for children born from 2009 onwards [25].

The incidence of overall IPD decreased gradually from 23/100,000 population in 2005 to 15/100,000 population in 2010 and 13/100,000 population in 2012, one year after PCV13 introduction (Table 1). The largest decrease was observed for PCV7-serotype IPD, particularly in children <2 years of age (incidence of 64/100,000 population in 2005 and zero in 2012). A similar decrease in other age groups (2–4 years, 50–64 years and ≥65 years) was also observed [25].

In 2005, PCV7 serotypes accounted for about 63 % of all IPD cases in children <5 years of age. In 2010, with the virtual elimination of all PCV7 serotypes (one case of 19 F reported), PCV13 serotypes comprised 61 % of IPD cases; serotypes 19A, 1 and 7 F were the most prevalent. In 2012, PCV13 serotypes still accounted for 26 % of all IPD cases although no cases of PCV13-serotype IPD were observed in children <2 years of age (Table 2). In those >5 years of age, PCV7 serotypes comprised 53 % of IPD cases in 2005, which gradually declined to 8 % in 2012. PCV13 serotypes accounted for 52 % of all IPD cases in 2010, decreasing to 45 % in 2012 (Table 3). Since 2010, IPD due to serotypes 23B and 15A increased. The number of cases caused by serotype 1 fluctuated between 1994 and 2002, became stable and then declined in all ages after the introduction of PCV13. In adults ≥65 years of age, a decrease in all PCV13 serotypes was observed with the exception of serotype 3 [25].

#### Spain (Madrid region)

PCV7 became available in 2001 and was incorporated into the regional immunization programme in Madrid in November 2006 with a 2 + 1 schedule and PCV13 was replaced in June 2010. Vaccine coverage was >90 % in the Madrid region [26].

In children <15 years of age, the incidence of IPD was significantly reduced from 17.1/100,000 population in 2007–2010 to 7.7/100,000 population in 2011–2012 (Table 1). In 2007–2009, non-PCV7 serotypes, such as 1, 19A, 5, 7 F and 3, became more prevalent. A significant reduction of PCV13 serotypes 1 and 19A was observed following PCV13 introduction in 2010 [27]. No cases of serotype 19A were observed in children <5 years of age in 2013. No significant change in the number of serotype 3 cases was observed, but there was an increase in serotype 8 cases. In 2013, serotypes 8, 3 and 19A were the most prevalent in the general population [26–28].

#### Switzerland

PCV7 was introduced into the NIP in November 2005 with a 2 + 1 schedule and was replaced by PCV13 in 2011. Vaccine coverage was around 76 % in 2012 with ≥2 doses in children <2 years of age [29].

From 2002–2005 to 2012, the overall IPD incidence decreased from 12.6 to 10.9/100,000 population. The most important decrease was in children <2 years of age, from 27.1 to 8.1/100,000 population. In adults >64 years of age, the decrease in IPD incidence was moderate and remained relatively high (42.1/100,000 population in 2002–2005 and 35.7/100,000 population in 2012) (Table 1) [29, 30]. In children <2 years of age, PCV7 serotypes accounted for 66 % of all IPD cases in 2004, which reduced to 17 % in 2010–2011 with the emergence of non-PCV7 serotypes, such as 3, 19A and 7 F. PCV13 serotypes comprised 68 % of IPD cases in 2010–2011, declining to 53 % in 2012, one year after PCV13 introduction. Serotypes 3 and 23 (not 23 F) remained the most prevalent serotypes in children, followed by 19 F (Table 2). In adults >64 years of age, PCV13 serotypes accounted for 66 % of all IPD cases in 2010–2011 and 62 % of cases in 2012. Serotypes 3, 19A and 7 F were the most prevalent in 2010–2012 (Table 3) [29, 30].

#### United Kingdom

PCV7 was introduced into the NIP in September 2006 with a 2 + 1 schedule and was replaced by PCV13 in April 2010. Vaccine coverage was >90 % in 2012–2013 [31].

The overall incidence of IPD significantly declined in all ages following PCV7 introduction in 2006 and PCV13 in 2010. From 2008–2010 to 2013–2014, the IPD incidence decreased from 22.2 to 12/100,000 population in children <2 years of age and from 27.6 to 20.6/100,000 population in adults >64 years of age (Table 1). In 2008–2010, non-PCV7 serotypes became prevalent, notably 7 F, 19A and 1 in children <5 years of age and 19A, 3 and 22 F in adults ≥65 years of age (Tables 1 and 2). The incidence of PCV7-serotype IPD reached near-extinction in 2013–2014 (0.20/100,000 population). Serotypes 6A/6C also significantly decreased in children <5 years of age.

Four years after the introduction of PCV13, the proportion of PCV13 serotypes decreased from 68 % (2008–2010) to 15 % (2013–2014) in children <5 years of age and from 54 % (2008–2010) to 21 % (2013–2014) in adults ≥65 years of age (Table 2). Serotypes 19A, 1, 7 F, 3 and 6A were significantly reduced, although year-on-year fluctuations were observed for serotype 3. Serotype 3 significantly decreased (44 % reduction) but remained prevalent in adults ≥65 years of age. Serotype 5, which is an outbreak strain, has not been detected in recent years. Non-vaccine serotypes generally increased in all ages and significantly in those >50 years of age. The most common serotypes were 24 F, 22 F and 33 F in children <5 years of age and 8, 22 F and 3 in adults ≥65 years of age (Tables 2 and 3) [10, 31].

### Countries in which only PCV10 is used

#### Austria

In 2012, PCV10 was the first PCV introduced into NIP with a 2 + 1 schedule. Vaccine coverage in 2012 was over 90 % [32].

Annually, about 200 to 330 IPD cases were reported to the national reference centre between 2009 and 2013 [33–37]. From 2011 to 2013, the total reported number of IPD cases in children <5 years of age was stable, with 14 reported cases per year (compared with 22 cases each in 2009 and 2010). PCV10 and PCV13 serotypes comprised 41 and 73 %, respectively, of all IPD cases in 2009 and 71 and 93 %, respectively, of IPD cases in 2013 (Table 2). In adults >50 years of age, PCV10 and PCV13 serotypes accounted for 37 and 69 %, respectively, of all IPD cases in 2009 and 25 and 59 %, respectively, of IPD cases in 2013 (Table 3). From 2009, serotype 3 was the most prevalent serotype for IPD in all ages, fluctuating from 16 to 22 % of all cases. Other common serotypes were 14, 19A and 7 F. In 2013, 88 % of IPD cases caused by serotype 3 occurred in individuals >50 years of age while only one case was reported in children <5 years of age. Serotype 14 accounted for 7–11 % of all IPD cases between 2009 and 2013. Serotype 19A comprised 3 % of all IPD cases in 2009, increasing to 6–7 % in 2010–2013.

#### Finland

PCV10 was the first PCV in Finland, introduced in September 2010 with a 2 + 1 schedule. Vaccine coverage was estimated as 95 % in 2012–2013 [38].

From 2004 to 2010, the incidence of IPD in children <1 year of age ranged from 50 to 69/100,000 population and from 27 to 37/100,000 population in those >64 years of age. In children <1 year of age, the incidence/100,000 population gradually decreased after the introduction of PCV10 to 50.7 in 2010 and 11.2 in 2014. From 2010 to 2014, the overall IPD incidence in adults >64 years of age remained unchanged at 30–33/100,000 population due to an increase in non-PCV10-serotype IPD despite a significant reduction in PCV10-serotype IPD in all ages (Table 1) [39].

In children <5 years of age, PCV10 and PCV13 serotypes accounted for 84 and 92 %, respectively, of all IPD isolates in 2010. By 2014, PCV10 serotypes decreased significantly and accounted for 19 % of all IPD isolates reported. However, although the number of isolates was small, the three additional serotypes included in PCV13 increased, representing 51 % all isolates. The most prevalent serotypes in 2014 were 19A (30 %), and 3 (19 %) (Table 2). In adults >65 years of age, PCV10 and PCV13 serotypes accounted for 55 and 72 %, respectively, of all IPD isolates in 2010. By 2014, PCV10 serotypes had significantly decreased and accounted for 26 % of all isolates while the three additional serotypes included in PCV13 increased, comprising 34 % of all isolates (Table 3). The most prevalent serotypes in 2010 were 14 (15 %), 3 (10 %), 23 F (8 %); in 2014 these were 3 (17 %), 22 F (12 %) and 19A (11 %). Serotypes 3 and 19A have increased gradually over the last 5 years (2010–2014) with significant numbers of cases occurring in older age groups (>50 years of age) [39].

#### The Netherlands

PCV7 was introduced into the NIP in June 2006 with a 3 + 1 schedule and it was replaced by PCV10 in April 2011. In November 2013, vaccine schedule was reduced to 2 + 1 [40]. Vaccine coverage was high with ≥94 % of infants born between April 2006 and 2008 receiving full vaccination by the age of 2 years [41].

Introduction of PCV7 into NIP in 2006 led to a major decrease in IPD due to PCV7 serotypes vaccine-type from 7.4 per 100,000 per year in 2004–2006 to less than 1 per 100,000 per year in 2012–2014 [40]. According to data from nine sentinel laboratories, in 2006, PCV7 serotypes accounted for 48 % of all reported blood isolates, which reduced substantially to 17 % in 2010 and further decreased to 7 % in 2013. In 2010, PCV10 and PCV13 serotypes accounted for 42 and 60 % of all IPD isolates, and the most prevalent serotypes were 7 F, 8, 19A and 1, comprising 46 % of all IPD isolates (Table 3). Two years after PCV10 was introduced into the NIP, these serotypes remained the most prevalent: of all isolates, 17 % were serotype 8, 12 % were serotype 7 F, 10 % were serotype 19A and 8 % were serotype 3. The proportion of PCV10-serotype IPD isolates reduced to 28 % and the proportion of IPD isolates containing the three additional serotypes included in PCV13 remained the same (17 %). Since 2009, serotypes 8, 12 F, 19A, 3, 22 F have gradually increased. No substantial change in prevalence was observed for serotypes 7 F and 5 [41].

### Countries in which both, PCV13 and PCV10 are used

#### Germany

PCV7 was introduced in July 2006 as part of the NIP with a 3 + 1 schedule and was replaced by higher-valent vaccines (PCV10 and PCV13) in 2009. Vaccine coverage was estimated at about 98 % for the first dose but only 64 % for the booster dose in 2014 and PCV 13 was predominantly used with >90 % market share [42].

The overall incidence of pneumococcal meningitis decreased significantly in children <16 years of age from 1.5 to 0.9/100,000 population between 1997-2001 and 2010, respectively, with a further reduction to 0.7/100,000 population in 2012. This was driven by the reduction in the incidence of pneumococcal meningitis in children <2 years of age, from 7.7 (1997–2001) to 4.1 (2010) and 3.7/100,000 population (2012). A substantial reduction was observed in non-meningitis IPD, most importantly in children <2 years of age (Table 1) [42, 43].

In children <16 years of age among pneumococcal meningitis, PCV7 serotypes accounted for 65 % in 2005–2006 and reduced to zero in 2012–2013. PCV13 serotypes accounted for 83 % in 2005–2006, 56 % in 2008–2009 and reduced to 12 % in 2012–2013. Similar trend was observed in adults >16 years of age for PCV13 serotypes IPD: 68 % in 2005–2006, 56 % in 2009–2010 and 25 % in 2012–2013 (Tables 2 and 3) [44].

In children <16 years of age, the most prevalent serotypes were 14 and 18C in pre-PCV7 period, 7 F, 18C and 1 in post-PCV7 period and 7 F, 19A and 10A in 2010–2013. In the adult population (>16 years of age), the most prevalent serotypes during 1992–2006 were 23B and 14. During 2007–2009, serotypes 3 and 7 F accounted for 13 and 7 %, respectively, of all IPD cases, and in 2010–2013, after the introduction of higher-valent PCVs, serotypes 7 F, 23B and 3 accounted for 27 % of all IPD cases (Table 3). PCV13-serotype IPD significantly reduced while a diversity of non-PCV13 serotypes emerged in some age groups. Compared with the period following the introduction of PCV7, serotype 3 increased in children, albeit in very small numbers, and decreased in adults >16 years of age. A significant increase in serotype 23B was observed for all ages [42, 44].

#### Sweden

PCV7 was introduced in January 2009 as part of the NIP with a 2 + 1 schedule and was replaced by higher-valent vaccines (PCV10 and PCV13) in 2010. Both higher-valent PCVs are currently used in the immunization programmes of different Swedish counties. PCV coverage in children <2 years of age was 98 % in 2012 and 2013 [45].

The overall IPD incidence ranged from 14.4 to 19.5/100,000 population during 2005–2011. Compared with the average IPD incidence in 2005–2007, incidence in 2012 decreased by 75 % and 57 % in children aged <2 years and 2–4 years of age, respectively. Among adults, no major changes were observed. In 2008, PCV7 serotypes accounted for 68 % of all IPD cases in children <2 years of age and this gradually decreased to 12 % in 2012. In 2009 (before the introduction of higher-valent PCVs), PCV13 serotypes accounted for 84 % of all IPD cases in children <2 years, which reduced to 47 % in 2012 (Table 2). In addition, there was a reduction in the proportion of IPD caused by PCV13 in all age groups (74 % in 2009 to 48 % in 2012). Serotypes 22 F, 7 F, 3 and 19A were the most common serotypes in 2011 and 2012. Serotypes 33 F and 11 became important in 2012 [45].

#### Spain (Catalonia, Navarra)

In Catalonia and Navarra, PCV7 became available in 2001 in the private market with a 3 + 1 schedule. PCV13 and PCV10 became available in 2010. Vaccine uptake was around 50 % in Catalonia and 78 % in Navarra in 2013. PCV13 was the most commonly used vaccine [46, 47].

In Catalonia, PCV7 serotypes accounted for 21 % of IPD cases in 2005, which reduced to 13 % in 2009. 19A was the most prevalent serotype followed by 14 and 1 in children <2 years of age. Serotype 1 was the most prevalent serotype followed by 19A in children 2–4 years of age. During 2005–2009, the most common serotypes in adults >65 years of age were 1, 3, 19A and 14 [46].

During 2001–2003 in Navarra, the overall incidence of IPD was highest with 87.1/100,000 population in children <5 years and 33.6 in adults ≥65 years of age. It was significantly reduced to 18.7/100,000 in children <5 years of age and 25.0/100,000 in adults ≥65 years of age in 2010–2013. The reduction was driven by PCV7 and PCV13 serotypes, particularly in children <5 years of age. The incidence of PCV7-serotype IPD decreased from 42.9 to 0.7/100,000 population and that of additional PCV13-serotype IPD from 37.7 to 8.6/100,000 population from 2001–2003 to 2010–2013 (Table 1). During 2004–2009, PCV7- and PCV13-serotypes accounted for 9 and 78 % respectively, of all IPD cases in children <5 years of age and 19 and 72 %, respectively, of cases in those ≥5 years of age. The most predominant serotype was 19A, which reduced from 32 to 24 % following the use of PCV13 in children <5 years of age. The decrease was less prominent in those ≥5 years of age, from 14 % (PCV7 serotypes) to 11 % (PCV13 serotypes). Serotypes 19A, 3 and 7 F remained important from 2010–2013 [47].

#### Portugal

PCV7 became available in Portugal in 2001 in the private market with 3 + 1 schedule. Vaccine coverage was 75 % in 2008. In 2009 and 2010 PCV10 and PCV13, respectively, were made available and by 2010, PCV13 was the most commonly used vaccine [48, 49].

A decline in the overall proportion of PCV7-serotype IPD cases in children since 2002 has been observed. PCV7 serotypes accounted for 56 % of infections in 1999–2002 and decreased to 17 % in 2006–2008 [49]. Following the use of higher-valent PCVs in 2010, the incidence of overall IPD decreased in children, particularly in young children, from 52.1 (2008–2009) to 25.1/100,000 population (2011–2012) in children <1 year of age and from 31.6 (2008–2009) to 16.1/100,000 population (2011–2012) in children 1–2 years of age. These decreases were driven by reductions in PCV13-serotype IPD, from 43.6 (2008–2009) to 14.8/100,000 population (2011–2012) in children <1 year of age and from 28.0 to 9.1/100,000 population in children 1–2 years of age (Table 1) [48].

During 2006–2008, the most common isolates in children were 1 (24 %), 19A (20 %), 7 F (12 %), 14 (7 %) and 3 (6 %). Serotype 1 was associated with cases of empyema and occurred more often in older children and 19A occurred in younger children (<2 years of age) [49]. In 2008–2009, the most prevalent serotypes in children under 18 years of age remained the same: 1 and 19A followed by 7 F and 14, which decreased substantially in 2011–2012. Non-vaccine serotypes, notably 10A, increased in children.

In the adult population in 2008, PCV7-serotype IPD significantly decreased, accompanied by a significant increase in serotypes 1, 7 F and 19A. PCV13 serotypes accounted for 70 % of all IPD cases in 2008 and decreased to 54 % in 2011 which was mainly driven by decrease in prevalence of serotype 1 and 5. In 2009–2011, the most predominant serotypes were 3 (13 % of all isolates) followed by 7 F, 19A, 14, 1 and 8 [50].

## Conclusion

The epidemiology of pneumococcal diseases has significantly changed with the implementation of routine childhood pneumococcal vaccination programmes in Western Europe. The overall incidence of IPD has decreased (Table 1), mainly driven by significant decreases of vaccine-type IPD in individuals of vaccine-eligible age as well as decreases in older individuals not eligible for pneumococcal vaccination in countries with high vaccine coverage. After 7–10 years of PCV7 use, the incidence of PCV7-serotype IPD became very low in all ages with virtual elimination in children, particularly in countries with high vaccine coverage, such as Belgium, Denmark, France, Norway and the UK [4, 5, 11, 14, 20, 25, 31]. Non-PCV7 serotypes became more frequent, particularly 19A, 7 F, 3 and 1 some years after the introduction of PCV7 [4, 5].

With the introduction of higher-valent vaccines, a rapid and significant reduction of additional serotypes included in these vaccines was consistently observed. In countries that used PCV13, the proportion of PCV13-preventable IPD cases in children <5 years of age declined significantly from 61–73 % in the period after the introduction of PCV7 to 15–53 % in the period after the introduction of PCV13 (Table 2). This was mainly driven by a significant and rapid decline of serotypes 19A, 7 F, 1 and 6A in both vaccine-eligible ages and older age groups (indirect effect). However, despite significant direct and indirect protection, vaccine serotypes such as 19A, 7 F and 3 were still circulating and prevalent in children. These serotypes were particularly important in older age groups. The extent of reduction of vaccine serotypes in older population (indirect effect) was lesser in the early period after the introduction of PCV13 compared with later periods and in countries with lower paediatric vaccine uptake. After 1–2 years after introduction in France, Norway and Switzerland, PCV13 serotypes accounted for 42–62 % of all IPD while only 21 % in the UK at 4 years after PCV13 introduction (Table 3).

In countries that used PCV10, the proportion of PCV13-preventable IPD cases remained high (70–86 % in vaccine-eligible ages), with a predominance of serotype 19A and 3 (Table 2). While some post-marketing case control and cohort-based studies have suggested potential cross-protection of PCV10 against 19A IPD in young children [38, 51, 52], a decrease in IPD caused by serotype 19A in older children and in adult populations was not observed. In fact, serotype 19A became significantly and increasingly prevalent, particularly in older age groups in Finland, the Netherlands and Austria (Table 3). A similar trend was observed in New Zealand [53]. In children, the incidence of serotype 3 was very low and reported numbers remained unchanged in most countries. A case–control study in the UK suggested that the effectiveness of PCV13 for serotype 3 in all age groups was non-significant [54]. However, a case–control study in the US (3 + 1 schedule) had described a significant vaccine effectiveness of 76 % (95 % CI 13; 93) against serotype 3 [55] and relevant decreases in serotype 3 IPD were observed in larger observational studies in the UK and France [20, 31].

Serotype diversity generally increased, with diverse serotypes emerging depending on the country, time from introduction of higher-valent PCVs and the types of vaccine used. However, newly emerging non-PCV13 vaccine types were shown to be less invasive with low case-carrier ratio [56, 57] and no individual serotype has emerged in the same way as serotypes 19A and 7 F emerged after the introduction of PCV7. To date, serotypes 24 F, 22 F, 8 and 15A are becoming important in countries that use PCV13 and serotypes 19A and 3 in countries that use PCV10. In this review, otitis media and pneumococcal pneumonia and related serotypes were not discussed, despite the fact that the public health value of pneumococcal vaccination is largely based on the these diseases. However, given the diagnostic and ethical problems to obtain appropriate cultures for testing, the serotype epidemiology of non-invasive diseases is largely unknown. In this respect, nasopharyngeal carriage studies may serve as a surrogate to monitor the serotype evolution in non-invasive diseases, as mucosal pneumococcal diseases results from direct infection.

The knowledge of emerging vaccine serotypes is important to continuously monitor the risk-benefit profile of PCVs. The data presented here are also needed to develop next generation pneumococcal vaccines.

## Abbreviations

IPD: Invasive pneumococcal disease
PCV: Pneumococcal conjugate vaccine
PCV7: 7-valent pneumococcal conjugate vaccine
PCV13: 13-valent pneumococcal conjugate vaccine
NIP: National immunization programme
